# Diamond Converted into Coke

**Published:** 1847-12

**Authors:** 


*Diamond Converted into Coke.*—Dr. Faraday exhibited some diamonds which he had received from M. Dumas, which had, by the action of intense heat, been converted into coke.

In one case, the heat of the flame of oxide of carbon and oxygen had been used; in another, the oxy-hydrogen flame, and in the third, the galvanic arc of flame from a Bunsen battery of a hundred pairs. In the last case, the diamond was perfectly converted into a piece of coke, and in the others the fusion and carbonaceous formation were evident. Specimens, in which the character of graphite was taken by the diamond, were also shown. The electrical character of these diamonds was stated also to have been changed, the diamond being an insulator, while coke is a conductor.—*Transactions of Brit. Association.*—*Jour. Franklin Institute.*

